# Antinociceptive Effects of the Serotonin and Noradrenaline Reuptake Inhibitors Milnacipran and Duloxetine on Vincristine-Induced Neuropathic Pain Model in Mice

**DOI:** 10.1155/2014/915464

**Published:** 2014-02-23

**Authors:** Soh Katsuyama, Hiromu Aso, Akira Otowa, Tomomi Yagi, Yukinaga Kishikawa, Takaaki Komatsu, Tsukasa Sakurada, Hitoshi Nakamura

**Affiliations:** ^1^Department of Clinical Pharmaceutics, Tohoku Pharmaceutical University, 4-4-1 Komatsushima, Aoba-ku, Sendai 981-8558, Japan; ^2^Department of Pharmacology, Daiichi College of Pharmaceutical Sciences, 22-1 Tamagawa-cho, Minami-ku, Fukuoka 815-8511, Japan

## Abstract

Vincristine is an anticancer drug used to treat a variety of cancer types, but it frequently causes peripheral neuropathy. Neuropathic pain is often associated with the appearance of abnormal sensory signs, such as allodynia. Milnacipran and duloxetine, serotonin/noradrenaline reuptake inhibitors, have shown efficacy against several chronic pain syndromes. In this study, we investigated the attenuation of vincristine-induced mechanical allodynia in mice by milnacipran and duloxetine. To induce peripheral neuropathy, vincristine was administered once per day (0.1 mg/kg, intraperitoneally (i.p.)) for 7 days. Mechanical allodynia was evaluated by measuring the withdrawal response to stimulation with a von Frey filament. In vincristine-treated mice, mechanical allodynia was observed on days 3–28 of vincristine administration. A single administration of milnacipran (40 mg/kg, i.p.) or duloxetine (20 mg/kg, i.p.) had no effect on vincristine-induced mechanical allodynia. However, repeated administration of milnacipran (20 or 40 mg/kg, once per day, i.p.) or duloxetine (5, 10, or 20 mg/kg, once per day, i.p.) for 7 days significantly reduced vincristine-induced mechanical allodynia. These results suggest that chronic vincristine administration induces mechanical allodynia, and that repeated milnacipran and duloxetine administration may be an effective approach for the treatment of neuropathic pain caused by vincristine treatment for cancer.

## 1. Introduction

Peripheral neurotoxicity induced by antineoplastic drugs (vinca-alkaloids, taxanes, and platin-based compounds) is a clinically significant complication that can be dose limiting and can substantially diminish the quality of life. Vincristine is a chemotherapeutic agent that can be used in the treatment of many types of human cancer, including leukemias, lymphomas, and sarcomas [1, 2]. The antitumor action of vincristine is due to its binding to beta-tubulin, which leads to disorganization of the axonal microtubule cytoskeleton. However, peripheral neuropathy is a relatively common side effect of chemotherapeutic treatment with vincristine, sometimes greatly reducing patients' quality of life and their ability to perform activities of daily living [3].

Antidepressants and anticonvulsants have suppressive effects on neuropathic pain [4, 5], and antidepressants are widely used for the treatment of neuropathic pain. Indeed, antidepressants, particularly tricyclic antidepressants (TCAs), are regarded as first-line drugs for the treatment of neuropathic pain [4]. Recently, newer antidepressants have become available, the antidepressant function of which is better understood than that of TCAs; they work by selectively inhibiting monoamine reuptake, namely, serotonin (5-HT) and noradrenaline (NA). These serotonin/noradrenaline reuptake inhibitors (SNRIs) are also used clinically as a treatment modality for neuropathic pain [6, 7].

The antinociceptive effects of SNRIs have been examined using a variety of animal models of pain [8]. The SNRIs milnacipran and duloxetine have been shown to be efficacious for the prevention and/or reversal of pain in several preclinical models of acute and chronic pain in rodents. Yokogawa et al. demonstrated that milnacipran significantly attenuated late phase paw licking behavior in the formalin test [9] and also reversed mechanical allodynia in the chronic constriction injury model and spinal nerve ligation model of neuropathic pain [10–12]. In addition, milnacipran had an antiallodynic effect in the paclitaxel-induced neuropathic pain model [13] and the streptozotocin-induced diabetic neuropathy model [10] and potentiated the antihyperalgesic effect of tramadol [14]. For duloxetine, clinical studies have confirmed its efficacy against pain in diabetic patients [15–17]. *In vivo*, duloxetine significantly attenuated late phase paw licking behavior in the formalin test and also reversed mechanical allodynia in the spinal nerve ligation model of neuropathic pain [18]. Importantly, duloxetine has also been shown to alleviate allodynia in several inflammatory and neuropathic pain models [19–23]. However, the antiallodynic effects of milnacipran and duloxetine on vincristine-induced mechanical allodynia have not yet been investigated.

The purpose of the present work was to examine the efficacy and validity period of the SNRIs milnacipran and duloxetine on vincristine-induced neuropathic pain in mice.

## 2. Materials and Methods

### 2.1. Animals

Male ddY-strain mice (Japan SLC, Hamamatsu, Japan) weighing an average of 23–25 g at the time of testing were used in the experiments. Mice were housed individually in a colony maintained in a controlled environment (12 h light/dark cycle, room temperature 23°C, 50–60% relative humidity) and had unlimited access to food pellets and water. All behavioral experiments took place during the light period between 10:00 and 16:00 in a quiet room. The animals belonging to the various treatment groups (*n* = 10 per group) were tested in randomized order. All experiments followed the Guidelines on Ethical Standards for Investigation of Experimental Pain in Animals [24]. Additionally, the study was approved (number 13012) by the Committees of Animal Care and Use of Tohoku Pharmaceutical University.

### 2.2. Experimental Protocol

Vincristine sulfate (Oncovin; Nippon Kayaku Company, Tokyo, Japan) was administered intraperitoneally (i.p.) to mice at doses of 0.025–0.1 mg/kg, once per day for 7 consecutive days. Vincristine dosing was determined based on previous studies using mice [25]. Milnacipran hydrochloride or duloxetine hydrochloride (Wako Pure Chemical Industries, Ltd, Osaka, Japan) was administered i.p. to mice. All drugs were dissolved in physiological saline, in an injection volume of 0.1 mL/10 g body weight. In the vehicle control group, physiological saline alone was administered by the same schedule. Mechanical allodynia of the hind paw was assessed using von Frey filaments with 0.4 g bending force. Briefly, mice were placed individually in a plastic cage with a wire mesh bottom. After they had adapted to the testing environment for 60 min, the von Frey filament was pressed perpendicularly against the mid-plantar surface of the hind paw from below the mesh floor and held for 3–5 s with the filament slightly buckled. Lifting of the paw was recorded as a positive response. Stimulation of the same intensity (0.4 g filament) was applied to the point of bending 10 times to the hind paw at intervals of 5 s. Mechanical allodynia was designated as the percentage of withdrawal responses (% response) to stimulation of the hind paw. Testing was performed for 2 days before the start of the experiment to accustom the mice to the testing procedures.

### 2.3. Data Analysis

Data are presented as means ± S.E.M. The statistical significance of the differences between groups was determined using one-way analysis of variance (ANOVA) followed by Dunnett's test or two-way ANOVA followed by Bonferroni's test. The level of statistical significance was set at 5% (*P* < 0.05) in all experiments. Statistics were performed using GraphPad Prism software (Graph Pad Software, Inc., San Diego, CA).

## 3. Results

### 3.1. Time Course and Dose Response of Vincristine-Induced Mechanical Allodynia

**Figure 1:**
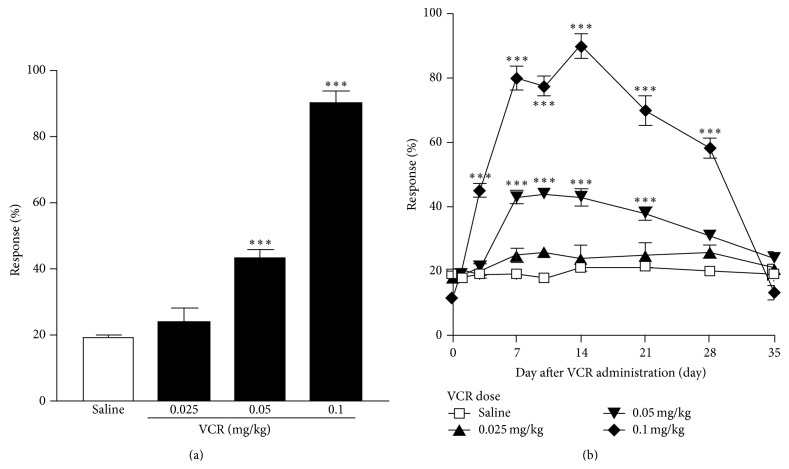
Vincristine (VCR; 0.025–0.1 mg/kg, i.p.) or saline was administered once per day for 7 days (days 0–6) to mice. (a) On day 14, the withdrawal responses to mechanical stimulation using a von Frey filament (0.4 g) were measured. (b) Time course of vincristine-induced mechanical allodynia. The withdrawal responses to mechanical stimulation using a von Frey filament (0.4 g) were measured on days 0 (Pre), 1, 3, 7, 10, 14, 21, 28, and 35, prior to daily vincristine administration. Data are presented as mean ± S.E.M. for 10 mice in each group. Significant differences between the groups were assessed with one- or two-way ANOVA followed by Dunnett's test (a) or Bonferroni's test (b), respectively. ^***^ *P* < 0.001 compared with the saline control group.

Vincristine or vehicle (saline) was administered to mice once per day for 7 days (days 0–6). We evaluated withdrawal responses to mechanical stimulation applied to the hind paw using a von Frey filament (0.4 g) from day 0 to day 35 in ddY mice. Mechanical allodynia was not observed when vincristine was administered i.p. at a dose of 0.025 mg/kg, as measured on day 14. However, when the dose was increased to 0.05 or 0.1 mg/kg, mechanical allodynia became marked (Figures 1(a) and 1(b)). With repeated daily dosing of 0.05 or 0.1 mg/kg of vincristine, mechanical allodynia peaked at day 14 after the start of administration and then decreased gradually, almost completely subsiding by day 35. In 0.1 mg/kg vincristine-treated mice, mechanical allodynia was significant on days 3–28 compared with the saline control group (Figure 1(b)). Thus, we subsequently used 0.1 mg/kg as the working dose of vincristine to induce mechanical allodynia. In contrast to the repeated dosing regimen, mechanical allodynia was not observed after a single administration of vincristine (0.05, 0.1 mg/kg; data not shown).

The effect of vincristine on the overall health of the mice appeared to be minimal. No mouse presented with weight loss or died within 35 days after administration.

### 3.2. Effects of Milnacipran or Duloxetine Treatment on Vincristine-Induced Mechanical Allodynia

**Figure 2:**
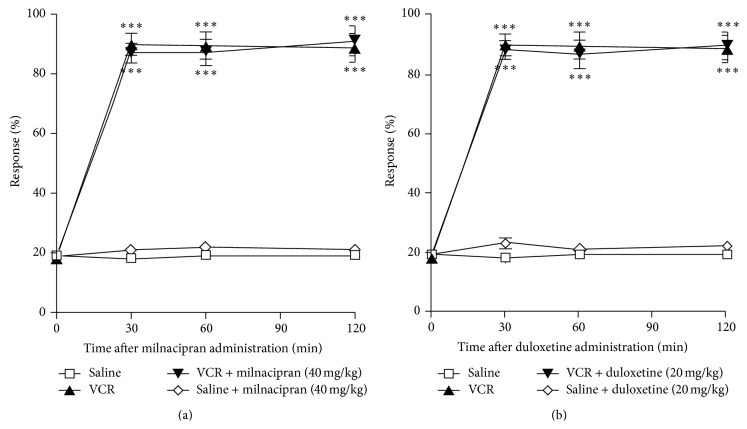
Vincristine (VCR; 0.1 mg/kg, i.p.) or saline was administered once per day for 7 days (days 0–6) to mice. On day 14, the withdrawal responses to mechanical stimulation using a von Frey filament (0.4 g) were measured at 0 min (Pre) and 30, 60, and 120 min after a single administration of milnacipran (40 mg/kg, i.p.) (a) or duloxetine (20 mg/kg, i.p.) (b). Significant differences between the groups were assessed with two-way ANOVA followed by Bonferroni's test. ^***^ *P* < 0.001 compared with the saline control group.

**Figure 3:**
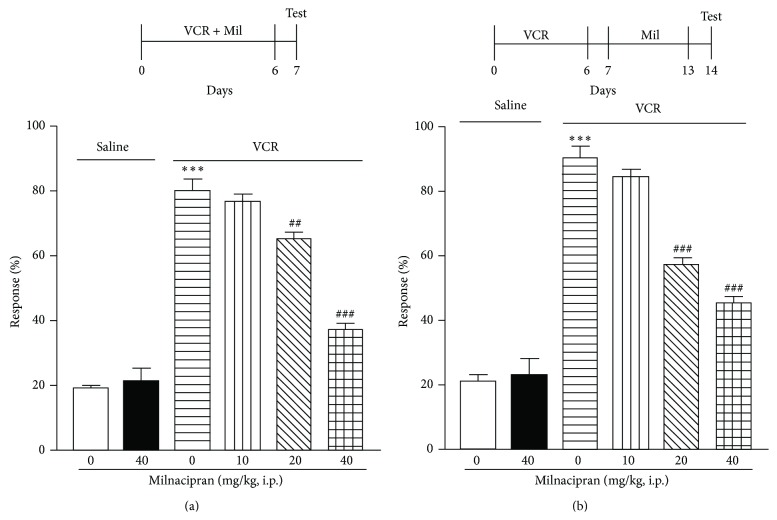
(Top) Schedule of drug administration. (Bottom) Vincristine (VCR; 0.1 mg/kg, i.p.) and milnacipran (Mil; 10, 20, or 40 mg/kg, i.p.) were coadministered once per day for 7 days (days 0–6) to mice (a). Milnacipran (Mil; 10, 20, or 40 mg/kg, i.p.) was administered to mice once per day for 7 days (days 7–13) after cessation of VCR (0.1 mg/kg, i.p.) administered once per day for 7 days (days 0–6) to mice (b). On day 7 (a) and day 14 (b), withdrawal responses to mechanical stimulation using a von Frey filament (0.4 g) were measured. Data are presented as mean ± S.E.M. for 10 mice in each group. Significant differences between the groups were assessed with one-way ANOVA followed by Dunnett's test. ^***^ *P* < 0.001 compared with the saline control group; ^##^ *P* < 0.01 and ^###^ *P* < 0.001 compared with the VCR group.

**Figure 4:**
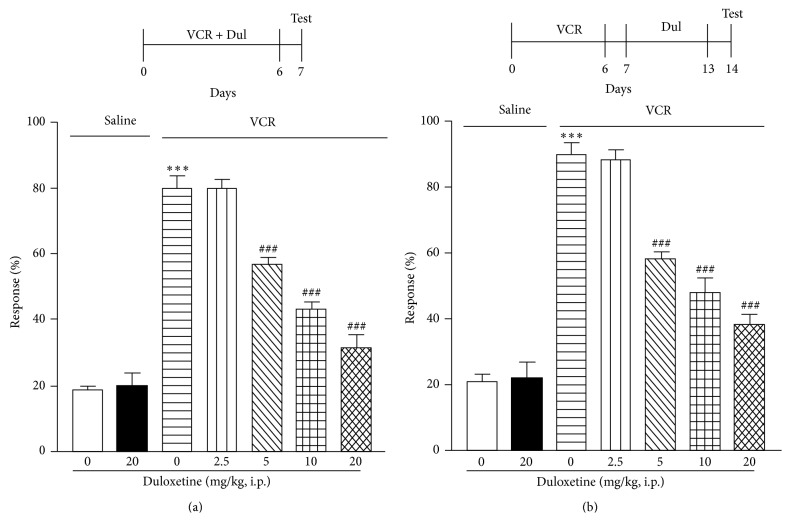
(Top) Schedule of drug administration. (Bottom) Vincristine (VCR; 0.1 mg/kg, i.p.) and duloxetine (Dul; 2.5, 5, 10, or 20 mg/kg, i.p.) were coadministered once per day for 7 days (days 0–6) to mice (a). Duloxetine (Dul; 2.5, 5, 10, or 20 mg/kg, i.p.) was administered to mice once per day for 7 days (days 7–13) after cessation of VCR (0.1 mg/kg, i.p.) administered once per day for 7 days (days 0–6) to mice (b). On day 7 (a) and day 14 (b), withdrawal responses to mechanical stimulation using a von Frey filament (0.4 g) were measured. Data are presented as mean ± S.E.M. for 10 mice in each group. Significance differences between the groups were assessed with one-way ANOVA followed by Dunnett's test. ^***^ *P* < 0.001 compared with the saline control group; ^###^ *P* < 0.001 compared with the VCR group.

A single administration of milnacipran (40 mg/kg, i.p.) or duloxetine (20 mg/kg, i.p.) on day 14, when allodynia peaked, had no effect on vincristine-induced mechanical allodynia (Figures 2(a) and 2(b)). However, repeated milnacipran administration (20 or 40 mg/kg, once per day, i.p.) or duloxetine administration (5, 10, or 20 mg/kg, once per day, i.p.) for 7 days (days 0–6), in combination with vincristine, significantly attenuated vincristine-induced mechanical allodynia on day 7 in a dose-dependent manner (Figures 3(a) and 4(a)). Similarly, when administered on days 7–13 (after cessation of vincristine treatment), these repeated dosing regimens significantly attenuated vincristine-induced mechanical allodynia on day 14 in a dose-dependent manner for milnacipran or duloxetine (Figures 3(b) and 4(b)). The single or chronic administration of milnacipran (40 mg/kg, i.p.) or duloxetine (20 mg/kg, i.p.) had no influence on the mechanical allodynia (Figures 2, 3, and 4).

## 4. Discussion

In this study, we demonstrated that repeated but not acute administration of vincristine (0.05 or 0.1 mg/kg, i.p.) produced dose-dependent mechanical allodynia in male ddY-strain mice. The mechanical allodynia was observed even after cessation of vincristine administration in this experimental model (Figures 1(a) and 1(b)). These observations are consistent with those of other reports of vincristine inducing mechanical allodynia in mice [25]. The present study further demonstrated a significant attenuation of vincristine-induced mechanical allodynia by repeated milnacipran or duloxetine treatment.

SNRIs such as milnacipran and duloxetine are used for the treatment of acute, persistent, neuropathic pain as supplementary analgesics [4, 8]. There have been several reports indicating that milnacipran and duloxetine have significant antinociceptive effects against nociceptive and inflammatory pain [9–13, 18–23, 26]. However, their efficacy for attenuating vincristine-induced mechanical allodynia remains to be established. In this study, repeated but not acute administration of the two agents significantly attenuated vincristine-induced mechanical allodynia, during both the development (days 0–6) and maintenance (days 7–14) stages of the allodynia.

5-HT and NA are implicated in modulating descending inhibitory pain pathways. Animal models of chronic pain have demonstrated the importance of serotonergic and noradrenergic systems [27]. The antiallodynic effects of SNRI in neuropathic pain models were predominantly attenuated by the noradrenergic system rather than by the serotonergic system at the spinal level [10]. Recently, Nakajima et al. reported that activation of the descending noradrenergic system and a subsequent increase of NA in the spinal dorsal horn in a rat neuropathic pain model contributed to the antihyperalgesic effects of antidepressants such as TCAs and SNRIs [28]. In addition, the *α*_2_-adrenergic receptor agonists significantly inhibited chronic constriction injury of sciatic nerve-induced neuropathic pain in mice [29], thermal hyperalgesia, and spinal astrocyte activation in a rat model of monoarthritis [30], further implicating adrenergic signaling in analgesia.

Central glial cells such as astrocytes and microglia play a prominent role in pain modulation [31, 32]. For instance, spinal astrocytes and microglia have been found to play critical roles in facilitating central plasticity following both nerve injury and inflammation [31, 32]. Indeed, spinal glial cells contribute to the development of vincristine-induced painful neuropathy in a mouse model [25]. Recently, Ji et al. reported that spinal astrocytes also contribute to the pathogenesis of vincristine-induced painful neuropathy in a rat model and also the neuropathy induced activation of the spinal astrocyte [33]. The neuronal NA transporter is functionally expressed in cultured rat astrocytes and SNRI potently inhibits NA uptake in these cells [34]. The NA uptake system in astrocytes is sensitive to milnacipran and duloxetine, and the therapeutic effects of SNRIs may thus be mediated to some extent by their action on astrocytes. Together with these findings, our data suggest that chronic but not single milnacipran or duloxetine treatment attenuates vincristine-induced neuropathic pain, that is, at least in part, through the inhibition of astrocyte activation.

## 5. Conclusion

These results suggest that chronic vincristine administration induces mechanical allodynia and that repeated milnacipran and duloxetine administration may be an effective approach for the treatment of neuropathic pain caused by vincristine treatment for cancer.
